# TP53-Deficient Angiosarcoma Expression Profiling in Rat Model

**DOI:** 10.3390/cancers12061525

**Published:** 2020-06-10

**Authors:** Urszula Smyczyńska, Damian Strzemecki, Anna M. Czarnecka, Wojciech Fendler, Michał Fiedorowicz, Marlena Wełniak-Kamińska, Magdalena Guzowska, Kamil Synoradzki, Łukasz Cheda, Zbigniew Rogulski, Paweł Grieb

**Affiliations:** 1 Department of Biostatistics and Translational Medicine, Medical University of Lodz, 92-215 Lodz, Poland; ulasmyczynska@gmail.com (U.S.); wojciech_fendler@dfci.harvard.edu (W.F.); 2 Department of Experimental Pharmacology, Mossakowski Medical Research Centre, Polish Academy of Sciences, 02-106 Warsaw, Poland; damian.strzemecki@gmail.com (D.S.); mfiedorowicz@imdik.pan.pl (M.F.); marlenak@imdik.pan.pl (M.W.-K.); magdalena_guzowska@sggw.pl (M.G.); ksynoradzki@imdik.pan.pl (K.S.); pgrieb@imdik.pan.pl (P.G.); 3 Department of Soft Tissue, Bone Sarcoma and Melanoma, Maria Sklodowska-Curie National Research Institute of Oncology, 02-781 Warsaw, Poland; 4 Department of Radiation Oncology, Dana-Farber Cancer Institute, Boston, MA 02284-9168, USA; 5 Small Animal Magnetic Resonance Imaging Laboratory, Mossakowski Medical Research Centre, Polish Academy of Sciences, 02-106 Warsaw, Poland; 6 Department of Physiological Sciences, Faculty of Veterinary Medicine, Warsaw University of Life Sciences, 02-776 Warsaw, Poland; 7 Faculty of Chemistry, Biological and Chemical Research Centre, University of Warsaw, 02-093 Warsaw, Poland; lcheda@chem.uw.edu.pl (Ł.C.); rogul@chem.uw.edu.pl (Z.R.)

**Keywords:** angiosarcoma, TP53, p53 TGEM Rat, microarray analysis

## Abstract

Sarcomas are a heterogeneous group of malignant tumors, that develop from mesenchymal cells. Sarcomas are tumors associated with poor prognosis and expected short overall survival. Efforts to improve treatment efficacy and treatment outcomes of advanced and metastatic sarcoma patients have not led to significant improvements in the last decades. In the *Tp53^C273X/C273X^* rat model we therefore aimed to characterize specific gene expression pattern of angiosarcomas with a loss of TP53 function. The presence of metabolically active tumors in several locations including the brain, head and neck, extremities and abdomen was confirmed by magnetic resonance imaging (MRI) and positron emission tomography (PET) examinations. Limb angiosarcoma tumors were selected for microarray expression analysis. The most upregulated pathways in angiosarcoma vs all other tissues were related to cell cycle with mitosis and meiosis, chromosome, nucleosome and telomere maintenance as well as DNA replication and recombination. The downregulated genes were responsible for metabolism, including respiratory chain electron transport, tricarboxylic acid (TCA) cycle, fatty acid metabolism and amino-acid catabolism. Our findings demonstrated that the type of developing sarcoma depends on genetic background, underscoring the importance of developing more malignancy susceptibility models in various strains and species to simulate the study of the diverse genetics of human sarcomas.

## 1. Introduction

Sarcomas, typically divided into soft tissue sarcomas (STS) and bone sarcoma (BS), are a heterogeneous group of malignant tumors, that develop from mesenchymal cells. Globally more than 130,000 new cases of sarcoma are diagnosed annually, and those make up 1% to 3% of the total number of all tumors in general. Sarcomas are burdened by poor prognosis with expected short overall survival. Only approximately 15% of patients with metastatic STS survive longer than 5 years. With combined treatment (neo-adjuvant chemotherapy and/or radiation therapy, radical surgery, and adjuvant chemotherapy), the five-year survival rate for patients with localized disease at diagnosis is within a range of 60–80% (depending on STS/BS histology). However, for poor chemotherapy responders and patients with initially metastatic disease, survival times are much shorter, with <50–30% and <10% five-year survival rates, respectively. Therefore, improvement of sarcoma treatment still represents an important medical challenge. Clinical outcomes of sarcomas have plateaued for the last 10 years and currently, anthracycline-based regimens, cyclophosphamide, vincristine, actinomycin-D, ifosfamide, etoposide and trabectidin are routinely used in treatment of metastatic disease. The only molecular discovery-driven targeted drug that is currently used is pazopanib—a tyrosine kinase inhibitor effective in selected STS, including angiosarcoma. Novel discoveries in sarcoma cell biology, genetics and genomics may contribute to identification of novel drug targets and eventually change management paradigms of STS, as was the case for some solid tumors, including breast or colon cancers [1,2,3,4]. 

Efforts to improve treatment efficacy and treatment outcomes of advanced and metastatic sarcoma patients have not led to significant improvements in the last several decades [3]. Currently there is still an urgent need to improve basic knowledge about sarcoma molecular biology and oncogenesis in order to accelerate the development of new therapeutic compounds that could potentially address this neglected area. A better understanding of the gene expression landscape observed in sarcomas is indispensable to the development of new effective therapeutic regimens or molecular profiling of individual cases in order to implement available drugs based on molecular tumor board decision [5,6]. 

*TP53* is the most frequently altered gene in cancers, with *TP53* mutations observed in approximately half of all tumors and more cases exhibiting epigenetic deregulation of *TP53* [7,8,9]. Inactivation of p53 plays a critical role in sarcomagenesis. It was shown that new germline mutations of the *TP53* gene, although rare among patients with sporadic STS, are, however, reported in patients with family history of sarcoma. A high rate of point mutations in *TP53* is reported not only in childhood sarcomas and families with the Li-Fraumeni syndrome, but also in adult-onset sarcomas, including leiomyosarcoma, osteosarcoma, and undifferentiated pleomorphic sarcomas [10,11]. It has also been described that alterations of *TP53* in rhabdomyosarcoma include a complete deletion of both *TP53* alleles, complete deletions of one allele with or without point mutation of the other allele, and absence of detectable transcript (mRNA). On the other hand, osteosarcomas are characterized with homozygous deletions of *TP53*, lack of *TP53* RNA expression or aberrant expression of p53 protein. The most recent genomic analysis has confirmed that, in angiosarcoma, the most common alterations are denoted as *TP53* mutation, *TP53* c.217-c.1178 missense substitution, and *TP53* missense (besides *MYC* amplification, and *KDR* mutation) [12]. All these data confirm that *TP53* functional inactivation by either dominant or recessive manner plays a significant role in human sarcomatogenesis including angiosarcoma development [4,13].

**Figure 1:**
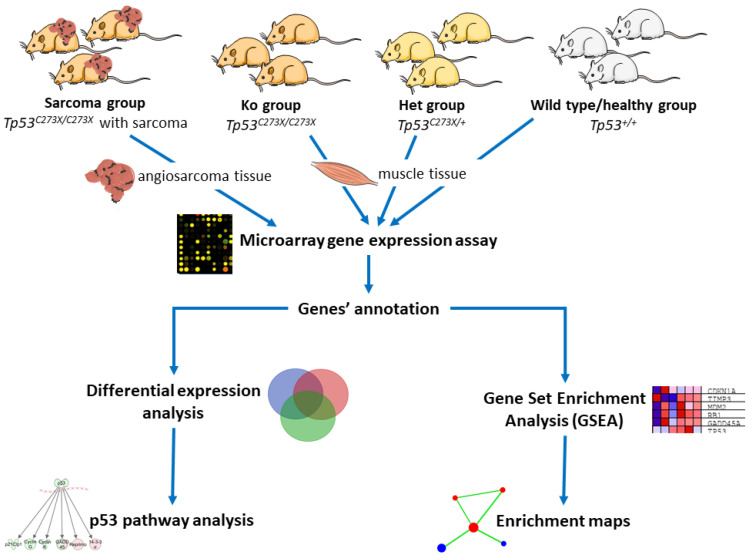
Roadmap for microarray gene expression analysis. Modified images from smart.servier.com [22] were used (available under Creative Commons license).

We used Wistar strain *Tp53* knockout rats (p53 TGEM^®^ Rat; TP53-deficient Wistar rat) *Tp53^C273X/C273X^* colony as a sarcoma development model. A single T to A point mutation in the *Tp53* DNA-binding domain introduces a premature C to X stop codon in position 273aa and is responsible for a loss of function of this tumor suppressor with no p53 protein detectable in the cells of knockout animals. Due to this nonsense mutation in the sixth exon, no full-length p53 is detectable in homozygous knockout rats and at the same time also no truncated protein is detectable in these homozygous rats, probably due to nonsense-mediated decay of its mRNA. In fact, complete absence of functional p53 protein in homozygous mutant animals was demonstrated already in embryonic fibroblasts [14]. Knockout rats develop angiosarcomas at four months of age at the latest. Surprisingly, it was shown that tumors from homozygous animals show very limited aneuploidy and low degrees of somatic copy number variation in comparison to the tumors that develop in heterozygous *Tp53^C273X/WT^* animals. In the tumors from knockout animals the complex structural rearrangements such as chromothripsis and breakage–fusion–bridge cycles were never found, despite being detectable in greater numbers in tumors from heterozygous animals. At the same time, in comparison to heterozygous tumors, tumors of knockout animals have longer telomeres but do not show clear telomerase activity or alternative lengthening of telomeres [15]. As it was previously shown in a mouse model, p53+/− animals are susceptible to oncogenesis and tumor development—due to a reduction in p53 dosage in cells [16]. All these data suggest that tumor development in *Tp53+/−* and *Tp53−/−* organisms can be driven by different mechanisms. Thus, we aimed to define specific gene expression patterns, related with *Tp53* loss of function (LOF)-driven sarcomatogenesis in a rat model. Actually, most LOF mutations in *Tp53* adhere to the two-hit hypothesis, as proposed by [17], and the most common cause of *Tp53* LOF is an inactivating missense mutation in one allele and simultaneous deletions in the regions of the 17p chromosome where the *Tp53* is located [18]. This animal model seems in fact clinically relevant as a high proportion (58%) of radiation-induced sarcomas exhibit a somatic inactivating mutation for one *Tp53* allele and a loss of the other. A high frequency (52%) of short deletions is observed in the mutation pattern of radiation-induced sarcomas [19]*; Tp53+^/C273X^* rats were recently used as model in the study on such tumors [20]. As we have shown before, *Tp53* knockout rats (p53 TGEM^®^ Rat) develop multiple tumors with angiosarcomas as the main tumor histotype [21]. Our experiments, therefore, aimed to characterize specific gene expression pattern of angiosarcomas with *TP53* LOF and represents a model that could be further used for pre-clinical drug testing and development, as well as imaging studies with novel agents. Briefly, we analyzed four types of samples: tumors (1) excised from *Tp53* homozygous knockout rats (‘Sarcoma’ group) or muscles excised from (2) from *Tp53* homozygous knockout rats (‘Ko’), (3) *Tp53* heterozygous knockout rats (‘Het’) and (4) wild-type healthy controls (‘Healthy’). The roadmap of the experiment is summarized in Figure 1.

## 2. Results

### 2.1. Tumors Localize in the Head and Neck, Extremities and Abdomen and Are Metabolically Active

**Figure 2:**
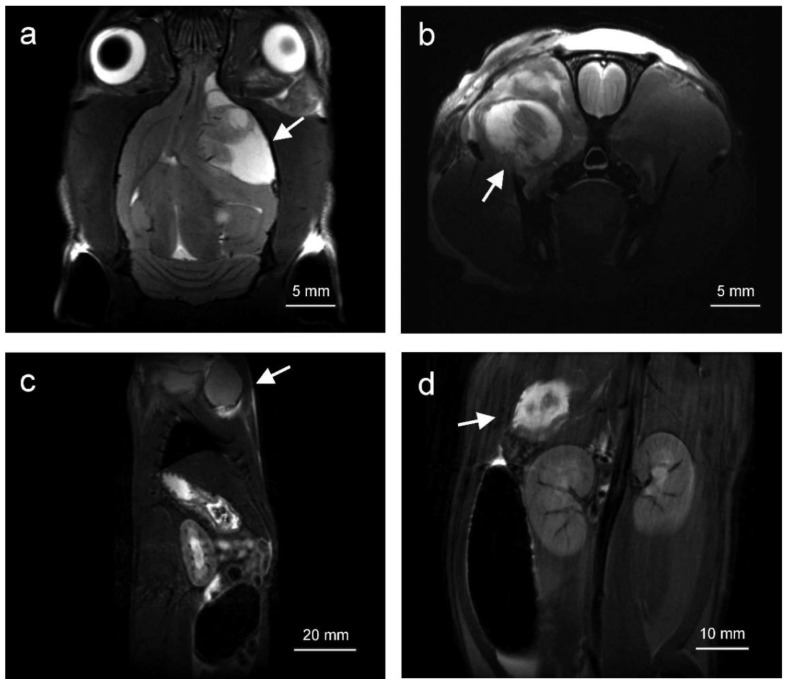
MR images depicting typical localization of tumors in *Tp53* homozygous knockout rats. Tumors were observed inter alia in the brain (**a**), skull muscles (**b**) as well as in the abdomen (**c,d**). Arrows indicate the tumors.

**Figure 3:**
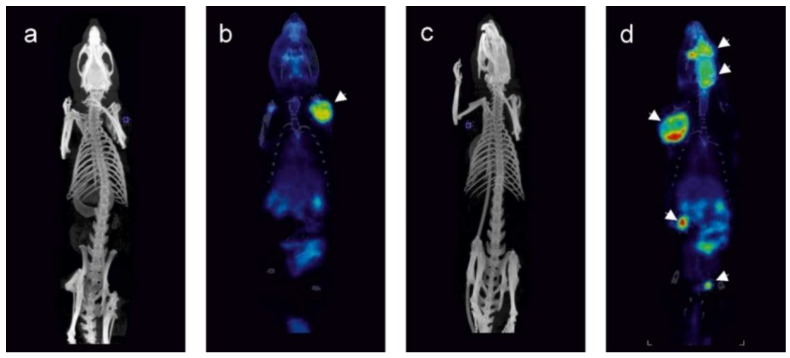
Representative FDG PET images (**b,d**) with reference CT scans (**a,c**) of tumor-bearing homozygous Tp53 knockout rats. Panels a and b illustrate a single tumor localized on the forelimb of the rat. Panels c and d illustrate a rat bearing several tumors: within the head, on the forelimb and within the abdomen. Tumors on PET images are indicated by arrows.

Solid tumors appearing in *Tp53* homozygous knockout rats (sarcoma group) were visualized by magnetic resonance imaging (MRI) and positron emission tomography (PET). The tumors were hyperintensive in T2-weighted magnetic resonance images (MRI, Figure 2). They were localized within the central nervous system, (Figure 2a), as well as within the muscles of head and neck (Figure 2b), soft tissues of extremities, abdominal cavity (Figure 2d) and dorsal muscles. The tumors were characterized by elevated 18F[FDG] uptake in comparison to adjacent tissues and clearly visible in positron emission tomography scans (Figure 3). Limb angiosarcoma tumors were selected for a genomic analysis. These tumor tissue samples presented upregulation of some genes typical for sarcoma (Figure S7).

### 2.2. Angiosarcoma Gene Expression Differ from Normal and Non-Sarcoma Tp53 Knockout Tissues

**Figure 4:**
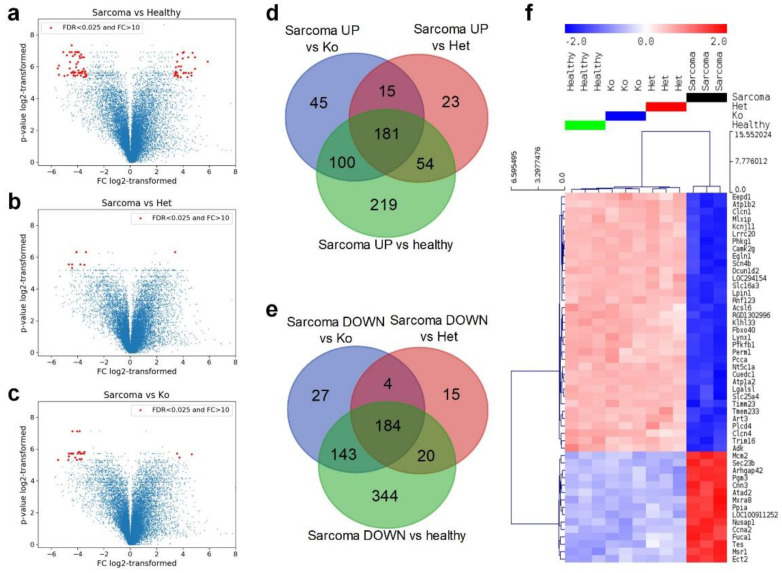
Comparison of gene expression and its overlap in four groups of rats. Volcano plots of gene expression in rats with sarcoma and all other groups: healthy (**a**), heterozygous knockout (**d**), total knockout (**c**). In panels (**a**–**c**), the horizontal axis presents log2-transformed fold change of gene expression, while the vertical axis presents log2-transformed *p*-value (after FDR correction). Venn diagrams of genes upregulated in sarcoma rats versus healthy controls and rats with heterozygous and total knockout (**d**), genes downregulated in sarcoma rats versus healthy controls and rats with heterozygous and total knockout (**e**). Hierarchical clustering of the top 50 differentially expressed genes (lowest FDR in ANOVA), Euclidean distance, linkage method: average, row-standardized (**f**).

In total, 3052 genes were differentially expressed in tissues of various groups of rats with the false discovery rate corrected *p* < 0.05 (referred as to FDR < 0.05), as shown by analysis of variance (ANOVA). Within this subset of genes, 1245 genes discriminated angiosarcoma and normal tissue. At the same time, expression of 496 gene-differentiated *Tp53* heterozygotes +/− and angiosarcoma while angiosarcoma and normal tissues of *Tp53* knockout −/− were discriminated by expression of 699 genes, both with FDR < 0.05. A large number of genes were differentially expressed in pairwise comparisons between angiosarcoma and all other groups (Figure 4a–c and Table 1).

The most extreme differences in gene expression (FC > 10 and FDR < 0.025, marked in red in Figure 4a–c) were observed between wild-type (*Tp53+/+*) normal tissue and angiosarcoma tissue in *Tp53−/−* rats. The 30 genes most strongly upregulated in angiosacroma were mostly involved in binding, structural and catalytic activities. Functions of 56 genes that showed the strongest downregulation in angiosarcoma included again binding and catalytic activity, and additionally transporter activity. Among the genes downregulated in sarcoma versus wild-type tissue, seven were identified as downregulated also in comparison of angiosarcoma tissue to all other groups, namely: muscle contraction and relaxation regulator; muscle chloride channel 1 (*Clcn1*), protein involved in ubiquitination; Kelch Like Family Member 33 (*Klhl33*), phosphohydrolase of triglyceride synthesis; Lipin 1 (*Lpin1*), myogenesis inhibiting myokine; Myostatin (*Mstn*), the enzyme responsible for dephosphorylation of nucleotides to nucleosides that regulates adenosine levels in muscles during ischemia and hypoxia-5′-Nucleotidase 1A (*Nt5c1a*), activator of the glycolysis pathway and an inhibitor of the gluconeogenesis pathway; 6-Phosphofructo-2-Kinase/Fructose-2,6-Biphosphatase 1 (*Pfkfb1*), responsible for lactic acid and pyruvate transport across plasma membranes; Solute Carrier Family 16 Member 3 (*Slc16a3*) (Table S2).

Overlap of genes significantly dysregulated in angiosarcoma versus all other tissues revealed 181 genes consistently upregulated in sarcoma and 184 downregulated ones (Figure 4d,e). At the same time, no genes were identified that differed in the inconsistent way, i.e., there were no genes significantly upregulated in sarcoma versus one group that were at the same time downregulated in sarcoma when compared with other ones (Table S3). Finally, all multiple comparisons between normal healthy tissue (*Tp53+/+*), with normal heterozygous *Tp53* knockout tissue (*Tp53+/−*), and normal knockout tissue (*Tp53−/−*), did not indicate any genes with significantly different expression. Only the angiosarcoma samples differed significantly from all other (normal) tissues. Moreover, typical angiosarcoma-related genes have shown an overexpression trend confirming the histopathologic profile of the analyzed tumors—tyrosine kinase with immunoglobulin-like and EGF-like domains 1 (*Tie1*), Fms-related tyrosine kinase 4, (*Flt4*), Fli-1 Proto-Oncogene (*Fli1*), ephrin type-A receptor 2 (*Epha2*), placental growth factor (*Pgf*), BHLH transcription factor (*Myc*) and Endothelin receptor type B (*Ednrb*) (Figure S8).

The results of principal component analysis (PCA) (Figure S1) indicated that angiosarcoma tissue samples can be separated from all other tissues on the basis of gene expression, while normal *Tp53+/+*, *Tp53+/−* and *Tp53−/−* tissues were very similar. Hierarchical clustering of the 50 top differentially expressed genes (lowest FDR) enabled separation of four tissue groups with a predominant difference between angiosarcoma and all other samples (Figure 4f), and indicating major changes in gene expression upon development of malignancy. In angiosarcoma cells, the most downregulated genes were: DNA binding protein; Endonuclease/Exonuclease/Phosphatase Family Domain-Containing Protein 1 (*Eepd1*), micro-satellite locus *Atplb2*, muscle chloride channel (*Clcn1*), channel regulating excitability of muscle tissues; potassium voltage-gated channel subfamily J member 2 (*Kcnjll*) and basic helix-loop-helix-leucine zipper transcriptional activator; Transcriptional Activator MondoA (*Mlxip*). At the same time, the most upregulated genes in angiosarcoma were DNA helicase, essential for genomic DNA replication: Minichromosome Maintenance Complex Component 2 (*Mcm2*), intracellular transport of proteins coat complex II component (*Sec23b*), sub-apical actin network organizing protein; Rho GTPase Activating Protein 42 (*Arhgap42*), glycogen metabolism regulator; Acetylglucosamine Phosphomutase (*Pgm3*) and promoting cell motility with stress fibers; Calponin 3 (*Cnn3*) (Figure 4f).

### 2.3. Angiosarcoma Presents Deregulated Metabolism

**Figure 5:**
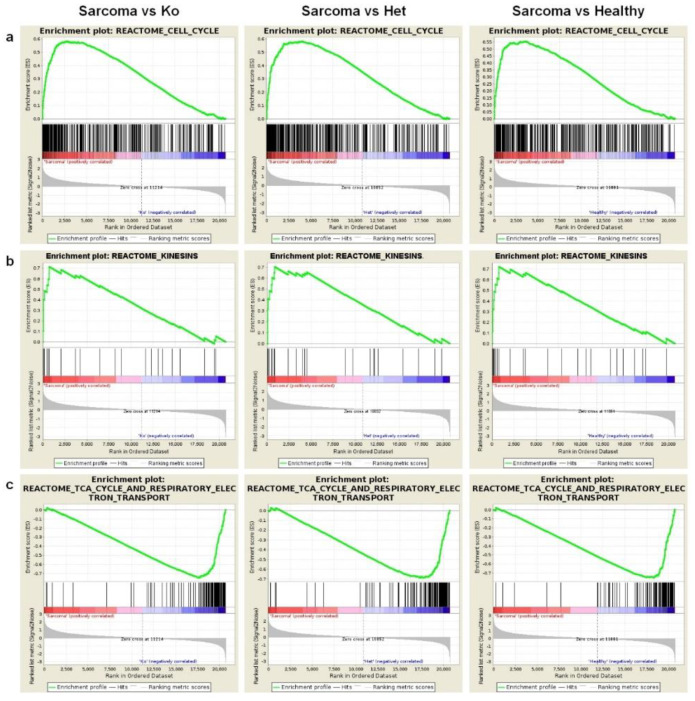
Gene set enrichment results of comparisons between the sarcoma group, healthy, heterozygous and homozygous *Tp53* knockout groups. (**a**) Enrichment plots of the Reactome cell cycle gene set that showed significant upregulation in the sarcoma group in comparison to the homozygous (left panel), heterozygous (middle panel) and healthy (right panel) groups. (**b**) Enrichment plots of the Reactome Kinesins gene set that showed significant upregulation in the sarcoma group in comparison to the homozygous (left panel), heterozygous (middle panel) and healthy (right panel) groups. (**c**) Enrichment plots of the Reactome TCA cycle and respiratory electron transport gene set that showed significant downregulation in the sarcoma group in comparison to the homozygous (left panel), heterozygous (middle panel) and healthy (right panel) groups.

Gene set enrichment analysis yielded results which corresponded to those obtained in a single gene analysis. The gene expression patterns of sarcoma samples differed strongly from all normal tissues with a number of gene sets which significantly differed in enrichment analysis. Cell cycle machinery genes (Figure 5a,b) were strongly upregulated in angiosarcoma samples. Conversely, citric acid cycle genes were strongly downregulated in angiosarcomas in comparison to all normal tissues (Figure 5c) as did glucose metabolism, pyruvate degradation, amino-acid catabolism and multiple other metabolic pathway gene sets (Table 2, Table S4). 

**Figure 6:**
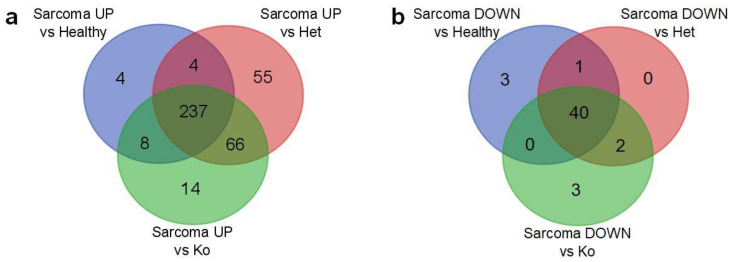
(**a**) Overlap of gene sets significantly upregulated in the sarcoma group in comparison to the healthy, heterozygous and total knockout groups. (**b**) Overlap of gene sets significantly downregulated in the sarcoma group in comparison to the healthy, heterozygous and total knockout groups.

**Figure 7:**
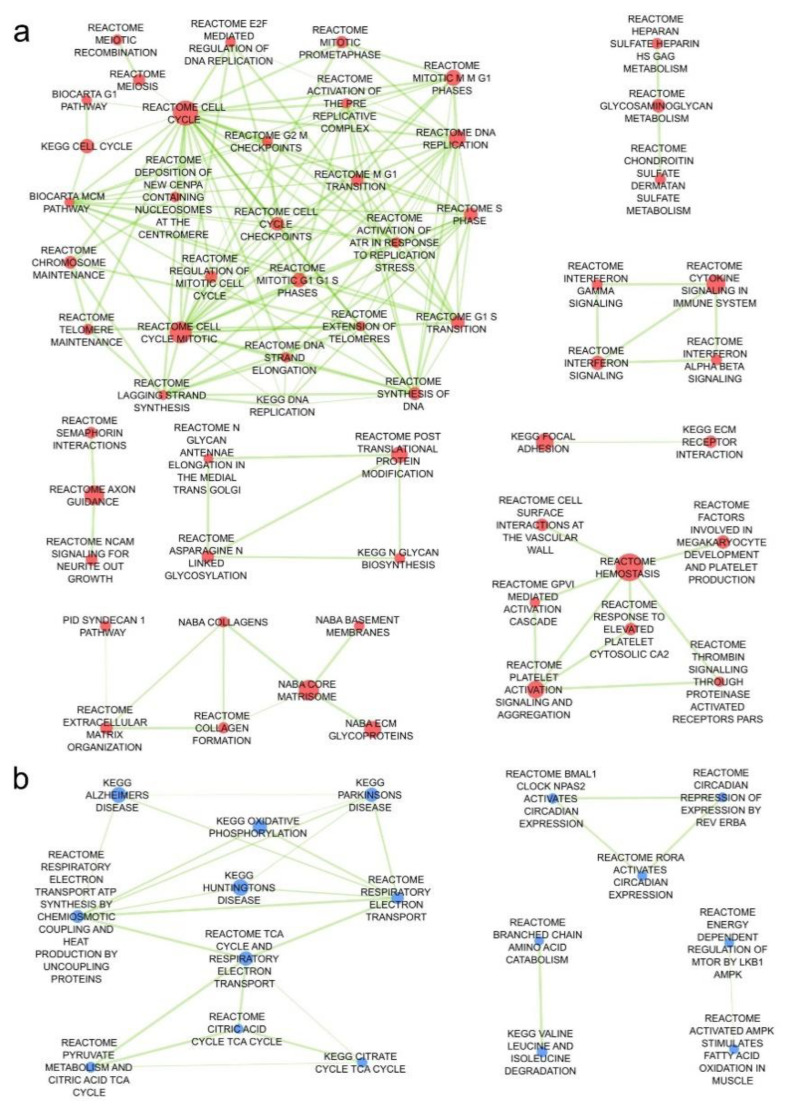
(**a**) Enrichment map of pathways significantly up or (**b**) downregulated in sarcomas versus the other three compared groups. Nodes (pathways) fulfilling filtration criteria, but without connections to other pathways are not shown (39 up- and 6 downregulated in sarcoma).

Next, we identified 237 up- and 40 downregulated pathways that were repeatedly deregulated in comparisons of angiosarcoma tissue versus all other normal tissues (Figure 6a,b and list in Table S5). The most upregulated pathways in angiosarcoma vs all other normal tissues were responsible for cell cycle including mitosis and meiosis, chromosome, nucleosome and telomere maintenance, as well as DNA replication and recombination (Figure 7a). As expected, sarcoma-related pathway genes have also been shown as upregulated including coagulation factor VIII (*F8*), Platelet endothelial cell adhesion molecule (CD31, *Pecam1*), marker of proliferation Ki-67 (*Mki67*), *Fli1*, thrombomodulin (*Thbd*), *Cd34*, tyrosine-protein kinase *Kit*, ETS transcription factor (*Erg*), podoplanin (*Pdpn*), mucin 1 (*Muc1*), cytokeratin Cam5.2, anion exchanger 1 (*Slc4a1*) and *Slc4a3* (Figure S7). Conversely, downregulated genes were responsible for metabolism, including respiratory chain electron transport, TCA cycle, fatty acid metabolism and amino-acid catabolism (Figure 7b). Curiously, a relatively low number of pathways were strongly dysregulated in angiosarcoma versus only one of the comparative groups (Figure 6a,b, Figure S2). Interestingly, angiosarcoma showed suppressed circadian regulation of selected biological processes in comparison to healthy and total *Tp53* (−/−) knockout tissues.

Among genes, significantly dysregulated in angiosarcoma (intersections of three circles in Venn diagrams in Figure 4d,e), 96 up- and 128 downregulated ones fulfilled the criteria of inclusion in drug discovery analysis. Finally, CMAP used 81 up- and 85 downregulated genes, while the rest were either identified but unused or unrecognized under any symbol available in NCBI gene database (list in Table S6). CMap analysis revealed six classes of pharmaceutical compounds (CMap classes) with enrichment score below −90.0, which means that their effect is highly opposite to the expression pattern observed in angiosarcoma samples. Those classes are: topoisomerase inhibitor (enrichment score −97.84), HDAC inhibitor (−95.95), CDK inhibitor (−94.38), DNA synthesis inhibitors (−92.69), JAK inhibitors (−92.60) and HIF activators (−91.06) (Table S7). The drugs predicted to have the highest activity were panobinostat (trade name Farydak by Novartis), ellipticine (currently not used in humans due to high toxicity), scriptaid (not yet developed for clinical applications), inducing transcription of p53 protein and blocking p53 ubiquitination—amsacrine (FDA approved in acute adult leukemia), PHA-793887 (currently not used in clinics due to severe dose-related hepatotoxicity), PF-562271 JNK-9L (a defined promising therapeutic target in phase 1 trial), bisindolylmaleimide-ix (drug candidate by Roche) and mitoxantrone trade name Novantrone and generics, FDA approved in hormone-refractory prostate cancer, acute myelogenous leukemia, breast cancer and non-Hodgkin’s lymphoma).

### 2.4. Activity of p53 Signaling Pathway Is Dysregulated in Tp53 Knockout Tissues

Ingenuity Pathway Analysis (IPA) revealed differences in expression of genes involved in P53 signaling between wild-type and *Tp53* knockout rats. In all knockout rats (sarcoma, ko and het groups) the *Tp53* gene itself is slightly, but insignificantly, downregulated (Figures S3–S5 and Table S2). The presence of *Tp53* transcripts, detectable by microarray assay, can be expected in this model, since loss of functional p53 is caused by point mutation, which results in the lack of p53 protein, but not necessarily lack of its mRNA. In the Het group majority, some—but not all—of the genes directly downstream of *Tp53* were downregulated, especially the ones responsible for DNA repair, which indicates that their activation by p53 was indeed suppressed. A similar transcriptomic profile was observed in Ko rats, in which, again, a substantial number of genes activated by p53 was downregulated in comparison to wild-type rats. In the sarcoma group, the activity of some of these genes seemed to be restored despite the presence of mutated *Tp53*. On the other hand, some genes responsible for cell cycle arrest were downregulated even in rats, which was consistent with our GSEA result that showed activation of cell cycle-associated pathways.

## 3. Discussion

Several p53-deficient animal models have been developed to mimic sarcomagenesis. In particular, mice with *Tp53,* inactivated by Cre-loxP-mediated recombination, develop spindle cell sarcomas and pleomorphic sarcomas. Additionally, mesenchymal sarcoma stem cells (Sca-1low) have been isolated from these animals [23]. The rat however is also a feasible model for imaging studies, easier in surgical handling and imaging than mouse. Unlike the *Tp53* knockout mouse that often develop lymphomas first, the *Tp53*-knockout rats most often develop sarcomas, which favors the use of the rat model in preclinical studies of sarcoma, including novel drug testing [14,24]. 

The first *Tp53*-deficient rat—Dark Agouti rat (subsequently referred to as the *Tp53*-deficient DA rat)—was created via homologous recombination in the rat embryonic stem cells. Homozygous *Tp53*-deficient DA rats live no longer than six months and develop angiosarcomas and lymphomas. Heterozygous *Tp53*-deficient DA rats survive up to 12 months of age and demonstrate a wide variety of sarcomas in both males and females, and also develop mammary carcinomas in about 20% of female rats [25,26]. On the contrary, in Fischer-344 (F344)’s rat-based 344-*Tp53^tm1(EGFP-Pac)Qly/^*^Rrrc^ (F344-*Tp53*) model, the tumor spectrum is shifted towards the primary tumor types—osteosarcomas and meningeal sarcomas. The incidence of osteosarcomas is 57% and 36% in F344-*Tp53* homozygous and heterozygous animals, respectively. In this model, tumors are highly representative of human disease radiographically and histologically. They typically localize on long bones and are characterized with frequent pulmonary metastases [7]. At the same time *Tp53* knockout rat in the Sprague Dawley background was generated using Zinc Finger Nuclease (ZFN) technology with target site located in the 22-bp exon 3 of the gene. A homozygous null rat—*Tp53^Δ11/Δ11^*—with a complete loss of p53 protein has a shortened disease-free lifespan due to early onset of cancers. The tumor spectrum in these null mutant rats includes both sarcomas and carcinomas, with a predominance of nervous system tumors. The *Tp53^Δ11/+^* rats experience a later onset of tumorigenesis and develop skin and endocrine cancers in addition to the cancer types recognized in the null homozygote [27]. Finally, the p53 TGEM Rat model that we used in this study was developed in a Wistar Han background (referred to subsequently as the *Tp53*-deficient Wistar rat). At the Hubrecht Institute, after N-ethyl-N-nitrosourea (ENU)-driven mutagenesis, a target-selected screen was performed in the outbred Wistar background. Adult male rats were administered intraperitoneal ENU injections that target spermatogonial stem cells (SSCs) in rat gonads. Animals with a nonsense mutation at amino acid position 273 (Cys to stop) within the DNA binding domain of the p53 protein were selected. This mutation functionally resulted in a full knockout *Tp53* mutation. For model development systematic generation of these knockout rats was carried out by random mutagenesis of Wistar rats followed by PCR amplification and capillary sequencing check-up, referred to as TGEM^®^ technology by Transposagen. ENU-driven target-selected mutagenesis is an effective approach for artificial introduction of point mutations. ENU, as an alkylating agent, transfers its ethyl group to oxygen or nitrogen in nucleophilic groups of nucleobases, which results in nucleotide substitutions such as A–T base transversions [28]. Wistar background homozygous mutant *Tp53^C273X/C273X^* rats predominantly develop sarcomas with an onset at four months of age and with a high frequency of pulmonary metastases. In our study, MRI and PET examinations revealed metabolically active tumors in several locations, including the brain, head and neck, extremities and abdomen. These sites were consistently similar to those previously described in this model [21]. Heterozygous rats develop sarcomas starting at eight months of age. *Tp53^C273X/+^* rats predominantly develop osteosarcomas, therefore this model may be generally used for soft tissue and bone sarcoma research and should be referred in appropriate papers, including imaging/PET-oriented manuals [28,29,30]. In the TGEM Rat model the introduced DNA mutation finally truncates the protein at the DNA binding domain, eliminating functionally essential domains including the nuclear localization domain and the homo-oligomerization domain of the translated protein, which results in rapid degradation of residual non-functional peptide. A genetic analysis confirmed that developed sarcomas in heterozygotes exhibit a loss-of-heterozygosity of the wild-type *Tp53* allele [14].

With regard to sarcoma-oriented studies, it must be pointed out that sporadic angiosarcomas (SA) and radiation-associated angiosarcomas (RAA) are similar in histology, immunohistochemical markers, and DNA mutation profiles and share a similar prognosis [31]. In angiosarcomas, most of abnormalities are found in the p53 and MAPK pathways. More than 50% of angiosarcomas presented MAPK pathway activation. Simultaneously, angiosarcoma genome analyses revealed mutations and amplifications of *VEGF*, *MDM2*, *TP53*, *CDKN2A*, *KRAS* and *MYC*. *TP53* was reported as mutated in 35% of the lesions and *CDKN2A* lost in 26%. Activating mutations were found in *KRAS*, *HRAS*, *NRAS*, *BRAF*, *MAPK1*, while inactivating mutations in *NF1* and *PTPRB1.* In particular, *MYC* gene amplifications are more common in RAA [32]. In our study, the most upregulated genes included Rho GTPase Activating Protein 42 (*Arhgap42*), mitochondrial Propionyl-CoA Carboxylase Subunit Alpha (*Pcca*), LOC294154 (similar to chromosome 6 open reading frame 106 isoform a) with ubiquitin binding activity, Spindle and kinetochore-associated protein 3 (*Ska3*) and Solute Carrier Family 16, Member 3 (Monocarboxylic Acid Transporter 4—*Slc16a3*). At the same time, the most upregulated pathways in angiosarcoma vs all other tissues were related to cell cycle with mitosis and meiosis; chromosome, nucleosome and telomere maintenance; as well as DNA replication and recombination. On the other hand, downregulated genes were responsible for metabolism, including respiratory chain electron transport, TCA cycle, fatty acid metabolism and amino-acid catabolism. Thus, the rat model that we studied here represents a novel interesting pre-clinical model that may easily be used for novel drug testing applications that surpasses a lack of tumor–host interactions and no immune response. This model has native microenvironment of an animal and also enables to conduct studies on an intact immune system response, including immunotherapy and cancer vaccines [33,34,35].

Our study analyzed the transcriptomic profile of *Tp53* knockout rats with either homo- or heterozygous point mutation in codon 273, both before and after tumor development. Future research can be aimed at verification if similar characteristics are observed at the proteomic level. The main limitations of our study are a relatively small number of animals and the fact that transcriptomic characteristics can change with time while we investigated them once in groups of rats of the same age. In our studies, Ko or Het rats without sarcoma were more similar in terms of transcriptome to wild-type animals than to those with the same mutation and developed tumor. We suspect that some changes may appear if such rats are observed for a longer time, which can be an interesting area of future research.

## 4. Materials and Methods

### 4.1. Animals

*Tp53* knockout rats (p53 TGEM^®^ Rat) with a Wistar strain genetic background (Charles River, Wilmington, MA, USA—transferred under exclusive license from Transposagen) were maintained as we described before [21]. A single T to A point mutation in the *Tp53* DNA-binding domain that introduces a premature C to X stop codon in position 273aa was present in *Tp53* gene with complete lack of p53 in homozygous mutant cells [14]. Genotyping of these animals was conducted as we described in detail previously [21]. In brief, DNA was extracted from a tail snip and extracted with a GeneMATRIX EURx kit (EURx Ltd., Gdansk, Poland). Genotypes of the rats were determined using simple allele-discriminating PCR (initial denaturation 94 °C for 6 min, 35 cycles of 94 °C for 20 s, 52 °C for 20 s, 72 °C for 20 s, final elongation 72 °C for 7 min) with two primer sets: (1) *Tp53* wild-type reverse primer (5′-GTCTCTCCCAGGACAGGTA-3′), and *Tp53* common forward primer (5′-GAAGACTCCAGGTAGGAAGC-3′) or (2) *Tp53* mutant reverse primer (5′-GTCTCTCCCAGGACAGGTT -3′) and *Tp53* common forward primer. An example of genotyping results is shown in Figure S6. Expression of p53 and CD31 was evaluated with Western blot (Figures S9 and S10). The tissue was pulverized by cryogenic grinding with liquid nitrogen and then lysed in RIPA buffer containing PMSF and cocktail of protease inhibitors (Sigma, St. Louis, MO, USA). Lysates were centrifuged at 14,000 rpm for 15 min. Protein concentration was measured using BCA assay (Pierce). In total, 20 µg of protein per lane was loaded on the gel. The antibodies used for Western blot analysis were as follows: mouse monoclonal anti-CD31 antibody (Merck MAB 1393Z, Darmstadt, Germany) and mouse anti-GAPDH (Chemicon MMAB374, Fisher Scientific, Waltham, MA, USA) and mouse anti-p53 (SantaCruz sc-126, Dallas, TX, USA). Secondary antibody was used as a goat anti-mouse IgG (Chemicon AP124P, Fisher Scientific, Waltham, MA, USA).

For the purpose of gene expression analysis, three rats in each of the four groups (Figure 1) were selected. The groups were the following: (1) Ko with sarcoma already developed (referred to as Sarcoma in figures and tables), (2) with total knockout of the *Tp53* (Ko), (3) with heterozygous knockout of the *Tp53* (Het), (4) healthy controls (Healthy). 

Animal research followed internationally accepted guidance for the care and use of laboratory animals, including the National Institute of Public Health—National Institute of Hygiene (NIPH–NIH) guidelines, and was approved by the IV Local Ethics Committee in Warsaw. All animal experiments were carried out in strict accordance with guidelines of IV Animal Ethical Committee in Warsaw (approval number 88/2015).

### 4.2. Magnetic Resonance Imaging

Magnetic resonance imaging (MRI) was performed to monitor the tumor growth in *Tp53* knockout rats. 7T Bruker Biospec scanner (70/30 USR, Bruker Biospin, Ettlingen, Germany) was used for the imaging and was equipped with: (1) a combination of a transmit cylindrical radiofrequency volume coil (8.6 cm inner diameter) with a rat brain dedicated receive-only array surface coil (2 × 2 elements)—for head and neck imaging or (2) a cylindrical radiofrequency volume coil (8.6 cm inner diameter) alone serving as a transmit-receive coil—for imaging of the abdomen. Animals were anesthetized with 1.5–2% isoflurane in oxygen, and positioned head first and prone in the MR-compatible animal bed. Respiration rate and body temperature were monitored throughout the experiment with a small animal monitoring system. Positioning tripilot scans were performed, followed by T2-weighted anatomical imaging. The head and neck region was imaged with TurboRARE T2 protocol (effective echo time, TEeff = 30 ms; repetition time, TR = 2500 ms; RARE factor = 8; flip angle, FA = 90°; 16 slices without gaps; field of view, FOV= 32 mm × 32 mm; spatial resolution=125 µm × 125 µm × 800 µm; number of acquisitions, NA = 4; time of acquisition, TA = 4 min). For abdomen imaging, the SNAP protocol was employed (TE = 2.6 ms, TR = 14 ms, FA = 25°, 25 slices, FOV = 80 mm × 100 mm, spatial resolution = 417 µm × 521 µm × 1000 µm, NA = 6, TA = 14 min).

### 4.3. [18F]FDG PET/CT

PET and CT scans were conducted with Albira PET/SPECT/CT Preclinical Imaging System (Bruker, Billerica, MA, USA) with spatial resolution of 1.5 mm for PET and 90 µm for CT. Animals were anesthetized with isoflurane (induction 4%, maintenance 1.5–2%) and 8–13 MBq of [18F]FDG (18F-Fluorodeoxyglucose, Gluscan 500, Advanced Accelerator Applications Sp. z o. o., Warsaw, Poland) was injected intravenously (in a total volume of 100–150 µL). PET/CT scans were started 60 min after [18F]FDG injection and reference CT scans were acquired (tube voltage 45 kVp, tube current 400 µA, number of frames 2, number of projections 600). PET and CT scans were fused using PMOD software, version 3.307, module Fusion Tool (PMOD Technologies LLC, Zurich, Switzerland).

### 4.4. RNA Isolation and Microarray Analysis

In healthy rats, biceps femoris was used for RNA isolation, and in the case of rats with sarcoma a tumor located within this muscle was used. Samples were excised and immediately frozen in liquid nitrogen and further stored at −80 °C (Revco Ultra Low–Temperature Freezer). RNA isolation was carried in a mortar bowl with RNeasy Fibrous Tissue Mini Kit (Qiagen, Hilden, Germany) as per manufacturer protocol with frozen muscle or tumor tissues. The GeneChip™ Rat Gene 2.1 ST Arrays and GeneChip WT PLUS Reagent Kit (Thermo Fisher Scientific, Waltham, MA, USA) were used to obtain expression data. 

### 4.5. Data Analysis

The experimental data, obtained for GeneChip™ Rat Gene 2.1 ST, were originally stored in 12 CEL files, each containing data about gene expression in a particular rat. AffySTExpressionFileCreator from GenePattern [36] was used to convert CEL files to numeric gene expression values. Additionally, background correction, log2-transform and quantile normalization were applied in conversion module. The gct file with expression data for 36,685 Probe IDs was created. Then, probe IDs were mapped to genes by ReannotateGCT module of GenePattern, according to probeset annotation file (release 36) downloaded from manufacturer website (Thermo Fisher Scientific, Waltham, MA, USA; www.thermofisher.com). Among 36,685 identified probesets, 21,527 were mapped to 20,740 unique rat genes. In cases when more than one probeset was mapped to a single gene, the highest expression among all those probes was selected for further analysis. Log2-transformed, quantile normalized gene expression data are available in Table S1. Pre-processed data were used in all further analyses.

First, gene expression in all groups of rats was compared by ANOVA, then obtained *p*-values were corrected by the False Discovery Rate (FDR) method [37] in order to find differentially expressed genes. Next, pairwise comparisons between particular groups were performed with the use of the *t*-test and FDR correction was applied for each pair of groups separately. Corrected *p*-value lower than 0.05 was considered significant. The PANTHER database was used for batch identification of molecular functions of differentially expressed genes [38]. Principal component analysis and hierarchical clustering (with Euclidian distance) were applied in identification and visualization of differences in gene expression profiles between the samples.

Then, gene expression profiles were subjected to Gene Set Enrichment Analysis (GSEA) [39]. The pathways from gene sets of C2CP (canonical pathways) from MSigDB [40] were analyzed as potentially affected by mutation and presence of sarcoma in rats. Since GSEA can compare only 2 phenotypes simultaneously, the 4 groups of rats were analyzed in pairs, giving 6 pairwise comparisons in total. GSEA allows to observe which pathways, not only single genes, are up- and downregulated in one group with respect to the other. Due to a low number of samples, gene set permutation method was used to estimate significance levels, which means that statistical significance of enrichment of particular gene set is calculated on the basis of gene expression in artificially created random gene sets of the same size. If the expression of the analyzed gene set is more extreme than in the majority (chosen fraction) of artificial gene sets, its enrichment is considered significant. In our analysis, we decided to use 1000 permutations to evaluate the enrichment in each gene set. Gene set size filter minimum and maximum were set to 15 and 1000, respectively. Additionally, GSEA was applied to examine expression of genes that are typically upregulated in sarcoma tissue [41].

The results obtained from GSEA were finally loaded into Cytoscape [42] in order to create Enrichment Maps (EM) that show the interconnectedness between the processes identified as abnormally active or suppressed. For comparisons between rats with sarcoma and any of the other 3 groups the *p*-value cut-off for EM was set to 0.001, FDR cut-off to 0.005 and overlap to 0.7, which means than only most significantly enriched pathways were retained in EM and the edges were drawn between pathways’ nodes when the number of overlapping genes was equal at least to 70% of size of the smaller of 2 gene sets. For clarity, gene sets without such overlap were excluded from figures.

The P53 signaling pathway was examined in Ingenuity Pathway Analysis (IPA). Our expression data were overlaid on the pathway scheme. This allowed us to compare expression of genes involved in P53 signaling between in 3 groups of knockout rats with wild-type animals. Moreover, angiosarcoma diagnostic genes have been evaluated to confirm histopathologic profile of the tumors including TIE1, FLT4, FLI1, EPHA2, PGF, MYC and EDNRB [43,44].

### 4.6. CMap Drug Discovery 

CMap [45] was used in order to identify compounds that could potentially reverse gene expression changes induced by angiosarcoma. The lists of genes most up- and downregulated in angiosarcoma versus all other groups were loaded into online CMap. Only genes significantly dysregulated (FDR < 0.05) in all pairwise comparisons between angiosarcoma and any of the other groups were taken into consideration. They were filtered by fold changed (FC > 4.0 for upregulation and FC < 0.25 for downregulation in all three comparisons of sarcoma rats vs others) so that the length of uploaded list did not exceed the CMap’s functional limit of 150 genes. In the cases when CMap did not recognize a gene symbol, all alternative symbols from the NCBI gene database were tested and the gene was marked as unrecognized when all of them were not identified by CMap. A list of perturbagens with their enrichment scores was retrieved from CMap. The scores can take values from −100 to 100 with positive sign meaning that particular perturbagen changes gene expression in similar manner to that observed in the analyzed experiment, and the negative sign indicating opposite action and absolute value showing the strength of the effect. In our experiment, the generated list of perturbagens was filtered to leave only compounds and CMap classes, while gene knockouts and over-expressions were omitted, since the study was aimed at in silico drug discovery. A detailed list of compounds from those groups with their individual enrichment scores below −90.0 was selected (Table 3).

## 5. Conclusions

In conclusion, the *Tp53^C273X/C273X^* rat model can be used for studying sarcoma biology, in particular angiosarcoma and osteosarcoma. It may be employed for evaluating new drug candidates and for screening novel compounds for their potential to induce sarcoma cell death, angiogenesis inhibition or anti-sarcoma immune response. Findings from this sarcoma model demonstrate that the developed type of sarcoma depends on genetic background, underscoring the importance of developing more new models in various strains and species to simulate the study of diverse genetics of human sarcomas [27,29,33,35].

## Figures and Tables

**Table 1:** Number of genes differentially expressed (FDR < 0.05) in pairwise comparisons between groups.

		Genes Upregulated In:			
		Healthy	Het	Ko	Sarcoma
**Genes Downregulated In:**	Healthy		0	0	554
	Het	0		0	273
	Ko	0	0		341
	Sarcoma	691	223	358	

**Table 2:** Number of gene sets significantly enriched in pairwise comparisons in GSEA.

		Pathways Upregulated In:			
		Healthy	Het	Ko	Sarcoma
**Pathways Downregulated in:**	Healthy		1	1	253
	Het	61		0	362
	Ko	5	0		325
	Sarcoma	44	43	45	

**Table 3:** Compounds belonging to classes identified by CMap as most opposing to gene expression changes resulting from angiosarcoma.

Compounds Groups	Enrichment Scores
topoisomerase inhibitor	−97.84
ellipticine	−98.37
amsacrine	−98.28
mitoxantrone	−97.89
amonafide	−97.85
topotecan	−97.72
teniposide	−96.72
SN-38	−96.44
irinotecan	−96.11
camptothecin	−95.88
doxorubicin	−95.42
pidorubicine	−95.28
daunorubicin	−95.14
pirarubicin	−94.78
HDAC inhibitor	−95.95
panobinostat	−98.45
scriptaid	−98.31
THM-I-94	−97.57
vorinostat	−96.79
belinostat	−96.72
apicidin	−95.00
trichostatin-a	−94.85
ISOX	−94.79
HC-toxin	−94.26
dacinostat	−92.65
givinostat	−91.73
CDK inhibitor	−94.38
PHA-793887	−97.96
JNJ-7706621	−97.43
AT-7519	−95.84
aminopurvalanol-a	−93.70
DNA synthesis inhibitor	−92.69
mitomycin-c	−94.37
JAK inhibitor	−92.60
JAK3-inhibitor-VI	−93.52
TG-101348	−92.57

## Supplementary Materials

The following are available online at https://www.mdpi.com/2072-6694/12/6/1525/s1, Table S1: Gene expression data (after log2-transform and quantile normalization), Table S2: Results of ANOVA and pairwise comparisons between different group of rats, Table S3: Lists of genes up- and -downregulated in angiosarcoma shown in Figure 3d,e, Table S4: GSEA results, Table S5: Lists of pathways up- and downregulated in angiosarcoma as shown in Figure 5, Table S6: List of genes used in CMap drug discovery analysis, Table S7: Results of CMap analysis for compounds, Figure S1: Principal component analysis of the microarray expression profiles with all 20740 genes included, Figure S2: Enrichment maps of pathways significantly change (**a**) in sarcoma versus healthy tissue only (unchanged both versus heterozygous and total knockout) (**b**) in sarcoma versus total knockout only, (**c**) in sarcoma versus heterozygous knockout only. Those maps were created as differences of enrichment maps for each pairwise comparison with nodes filtrated as in Figure 6 (that shows intersection of those same pairwise maps), Figure S3: Comparison of gene expression in P53 signaling pathway between rats with heterozygous knockout and wild-type animals. Green marks genes downregulated in Het group, red genes upregulated in Het rats, white genes not present in our dataset, Figure S4: Comparison of gene expression in P53 signaling pathway between rats with homozygous knockout and wild-type animals. Green marks genes downregulated in Ko group, red genes upregulated in Ko rats, white genes not present in our dataset, Figure S5: Comparison of gene expression in P53 signaling pathway between rats with sarcoma and wild-type animals. Green marks genes downregulated in sarcoma group, red genes upregulated in sarcoma rats, white genes not present in our dataset, Figure S6: An example of genotyping results. Product of PCR reaction with primer set designed to detect wild-type *Tp53* (wt) and mutated *Tp53* (mut) were run for each animal. Single wt band was interpreted as WT, single mut band—*Tp53−/−* and both bands were interpreted as *Tp53+/−*, Figure S7: Analysis of expression of genes related to sarcoma, that are typically upregulated, Figure S8: Analysis of expression of genes related to angiosarcoma, that are typically upregulated, Figure S9: Western blot analysis—p53 and CD31 expression. GAPDH expression was used as a control for protein load. SAR—sarcoma; CTL—positive controls, for CD31 rat white blood cells lysate (left lane) and homogenate from rat aorta (right lane); for p53 lysate from Caki1 cells (left lane) and MDA-MB-231 cells (right lane), Figure S10: Uncropped combined immunoblotting corresponding to Figure S9 with molecular weight markers and densitometry readings.
