# Supplementary material for: TP53-Deficient Angiosarcoma Expression Profiling in Rat Model
Source: Cancers (Basel). 2020 Jun 10;12(6):1525. doi: 10.3390/cancers12061525 (PMC7352674; doi:10.3390/cancers12061525)
Supplement: Supplementary file 1 [file cancers-12-01525-s001.zip › cancers-744721_supl-figures.pdf]

Supplementary materials: TP53 Deficient Angiosarcoma Expression Profiling in Rat Model

Urszula Smyczyńska, Damian Strzemecki, Anna M. Czarnecka, Wojciech Fendler, Michał Fiedorowicz, Marlena Wełniak-Kamińska, Magdalena Guzowska, Kamil Synoradzki, Łukasz Cheda, Zbigniew Rogulski and Paweł Grieb

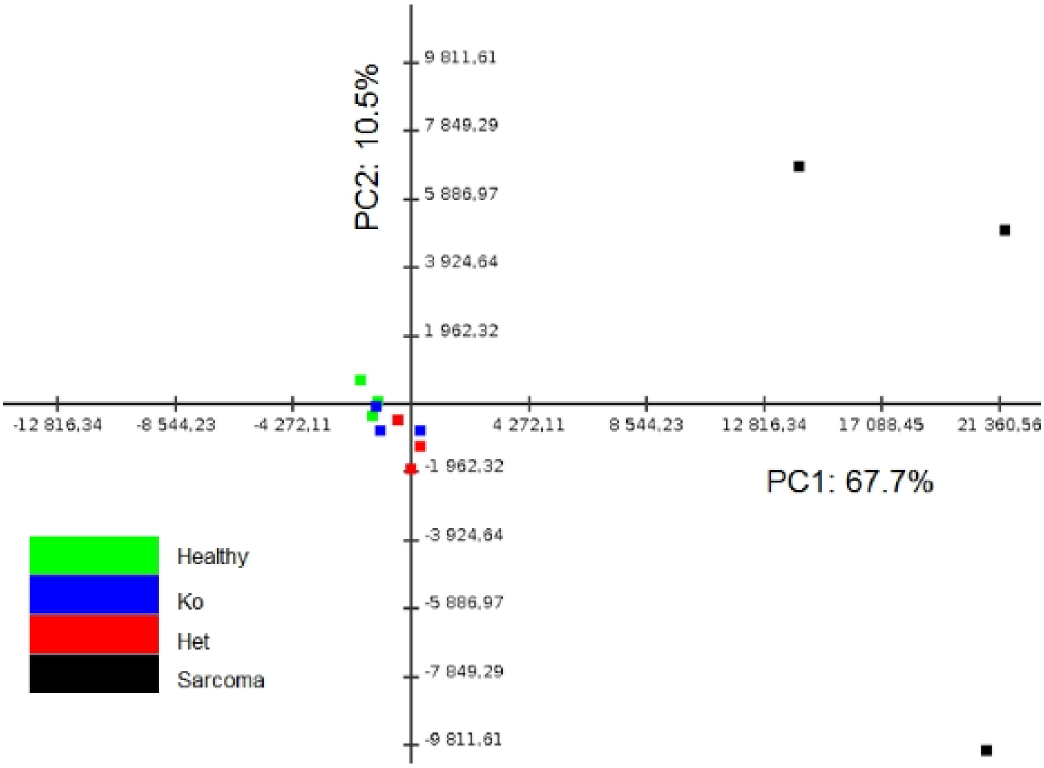

Figure S1. Principal component analysis of the microarray expression profiles with all 20740 genes included.

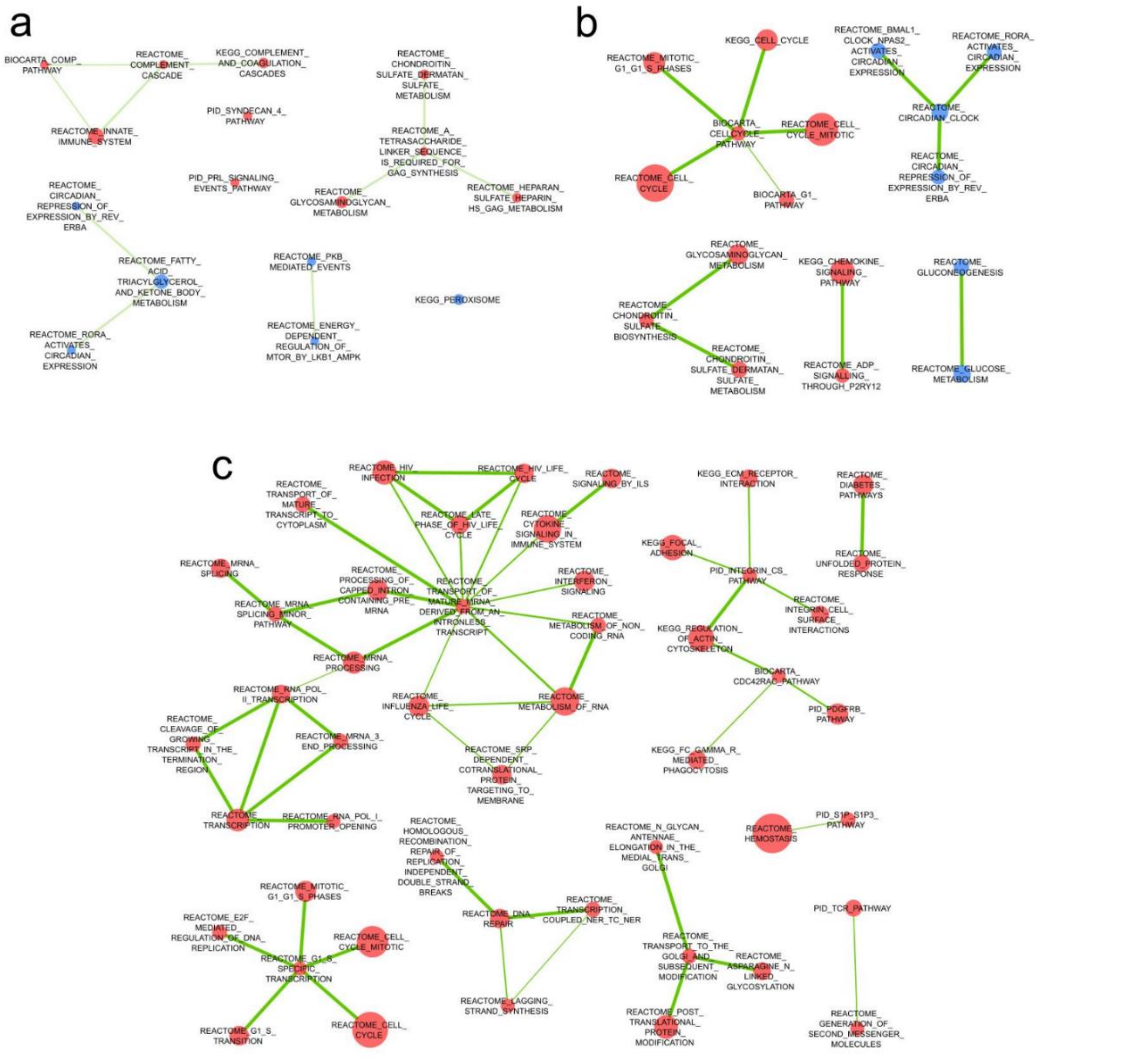

Figure S2. Enrichment maps of pathways significantly change **a**) in sarcoma versus healthy tissue only (unchanged both versus heterozygous and total knockout) **b**) in sarcoma versus total knockout only, **c**) in sarcoma versus heterozygous knockout only. Those maps were created as differences of enrichment maps for each pairwise comparison with nodes filtrated as in Figure 7 (that shows intersection of those same pairwise maps).

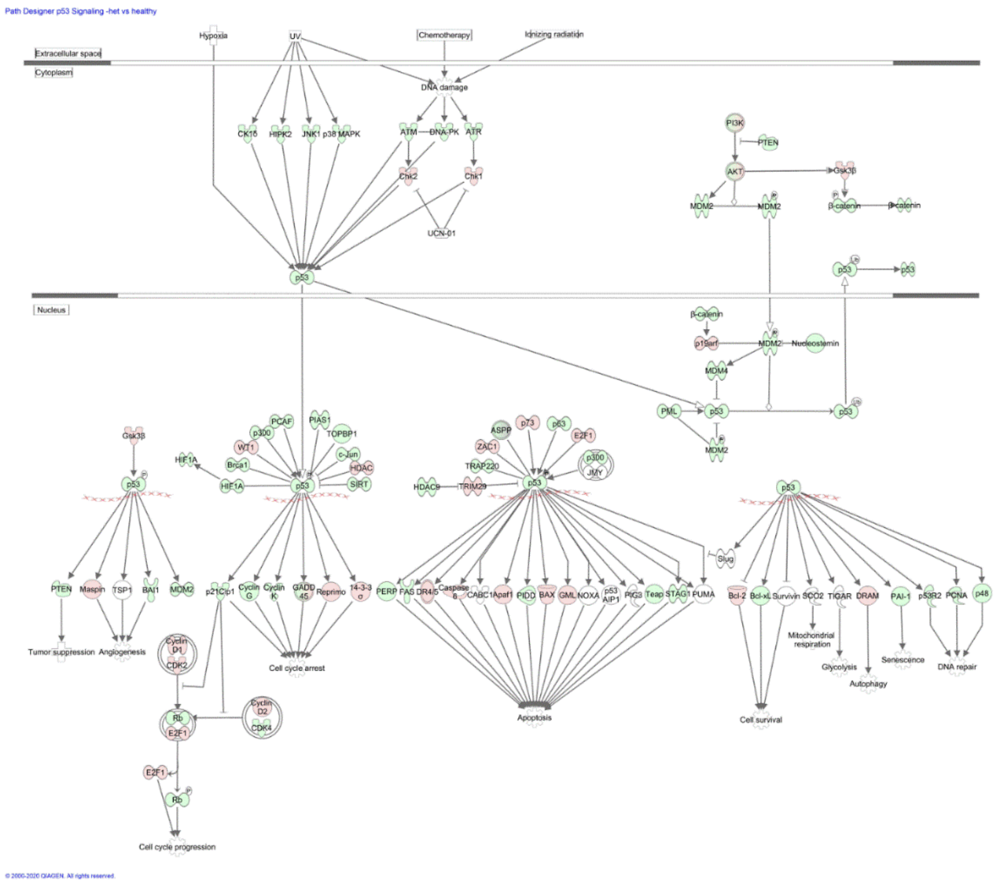

Figure S3. Comparison of gene expression in P53 signaling pathway between rats with heterozygous knock-out and wild type animals. Green marks genes downregulated in Het group, red genes upregulated in Het rats, white genes not present in our dataset.

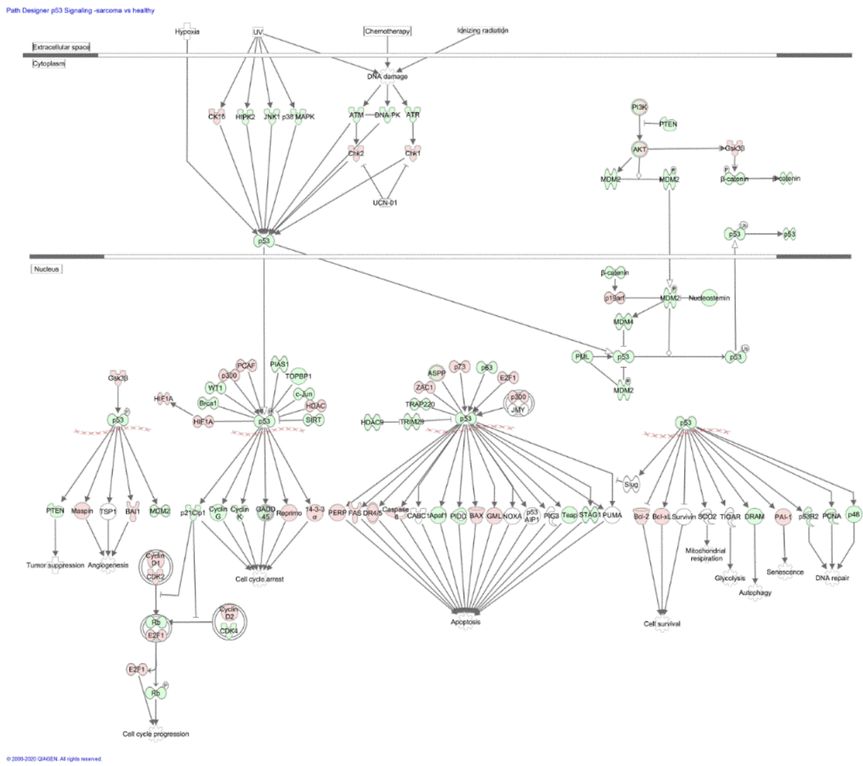

Figure S4. Comparison of gene expression in P53 signaling pathway between rats with homozygous knock-out and wild type animals. Green marks genes downregulated in Ko group, red genes upregulated in Ko rats, white genes not present in our dataset.

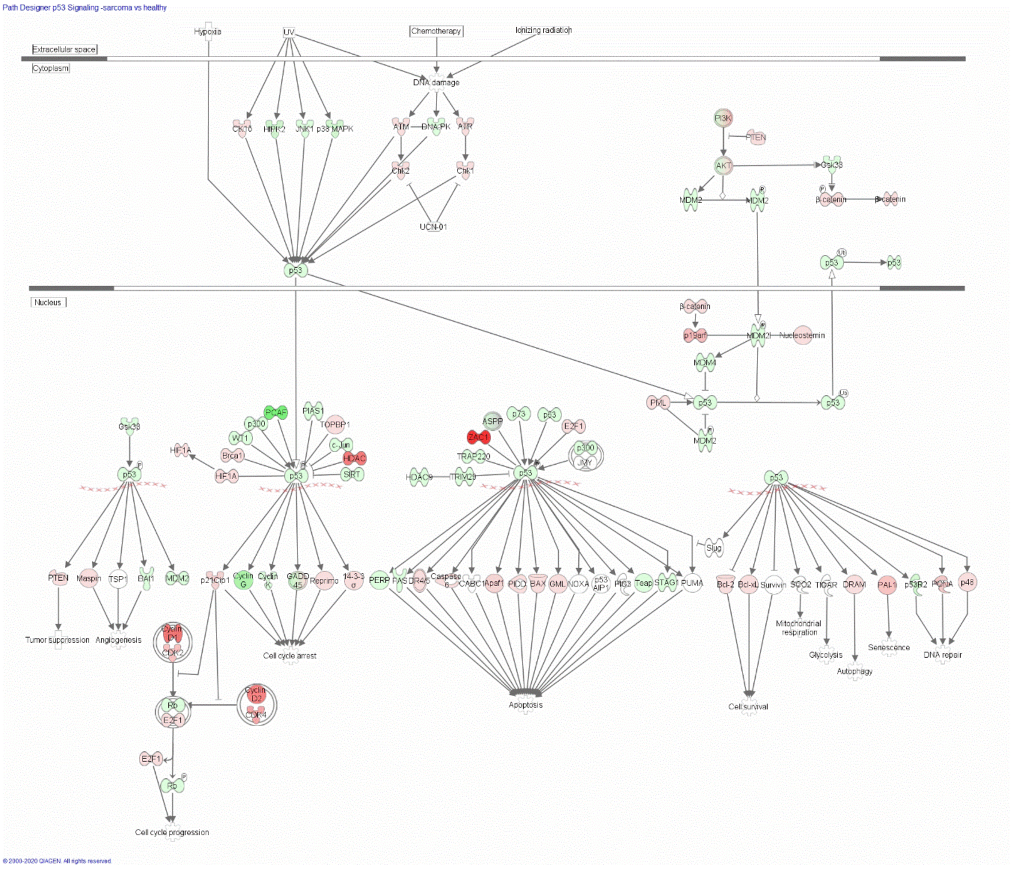

Figure S5. Comparison of gene expression in P53 signaling pathway between rats with sarcoma and wild type animals. Green marks genes downregulated in sarcoma group, red genes upregulated in sarcoma rats, white genes not present in our dataset.

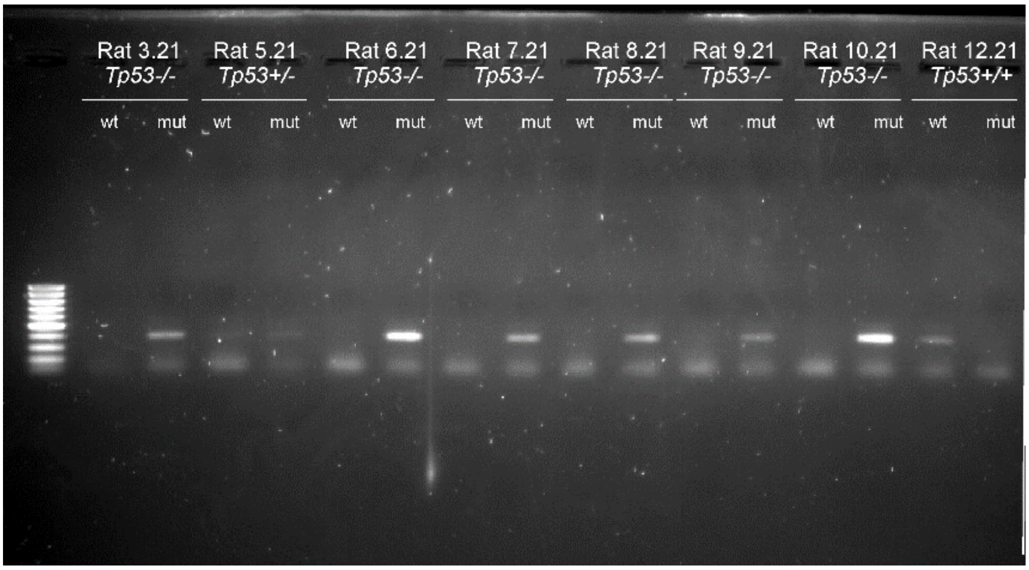

Figure S6. An example of genotyping results. Product of PCR reaction with primer set designed to detect wild type *Tp53* (wt) and mutated *Tp53* (mut) were run for each animal. Single wt band was interpreted as WT, single mut band—*Tp53*−/− and both bands were interpreted as *Tp53*+/−.

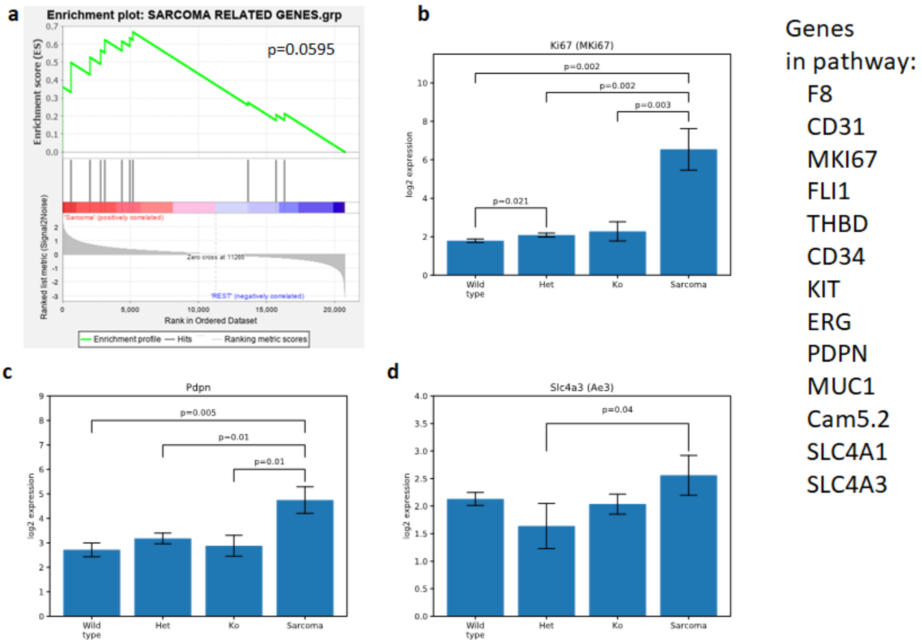

Figure S7. Analysis of expression of genes related to sarcoma, that are typically upregulated.

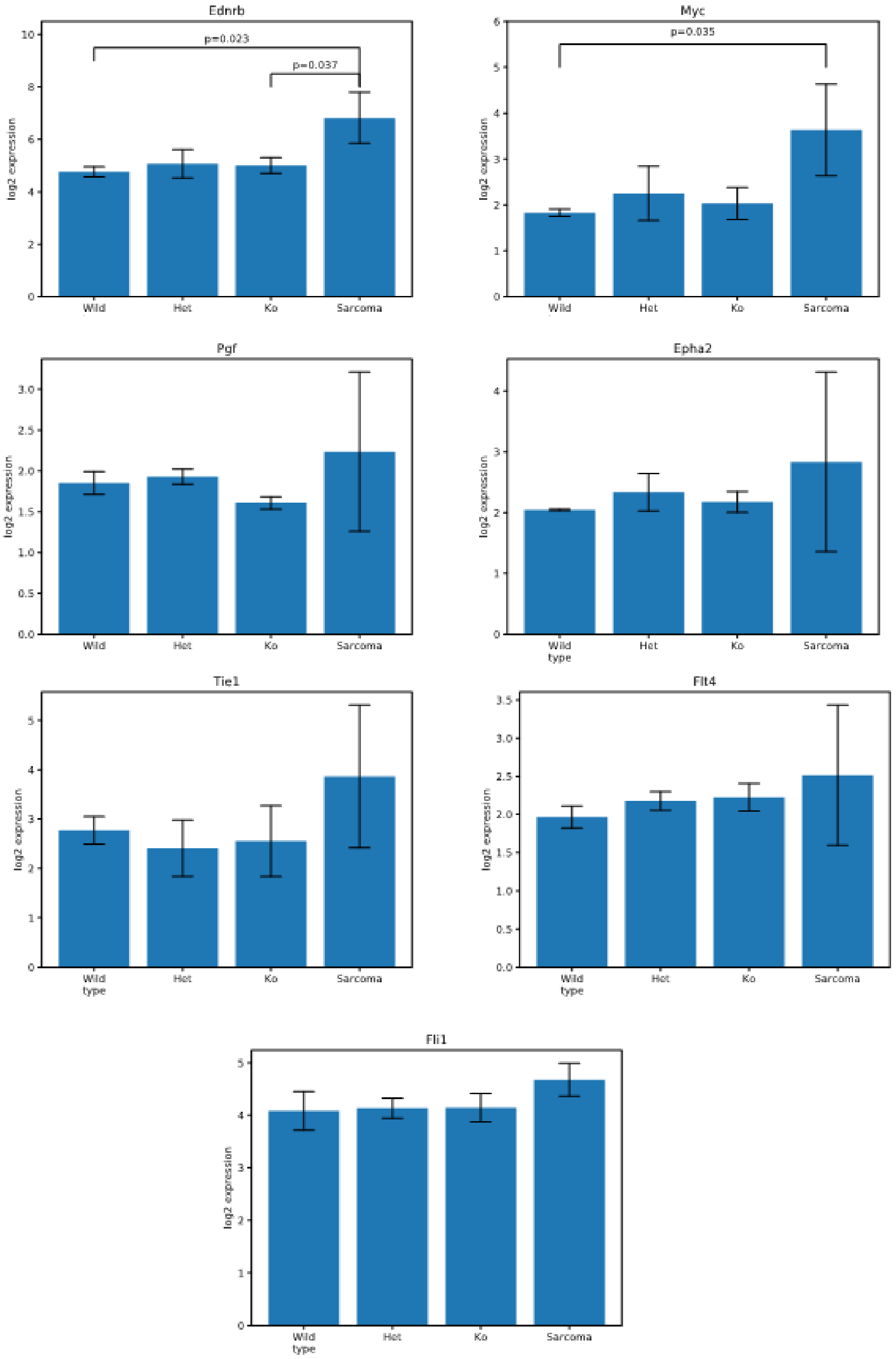

Figure S8. Analysis of expression of genes related to angiosarcoma, that are typically upregulated.

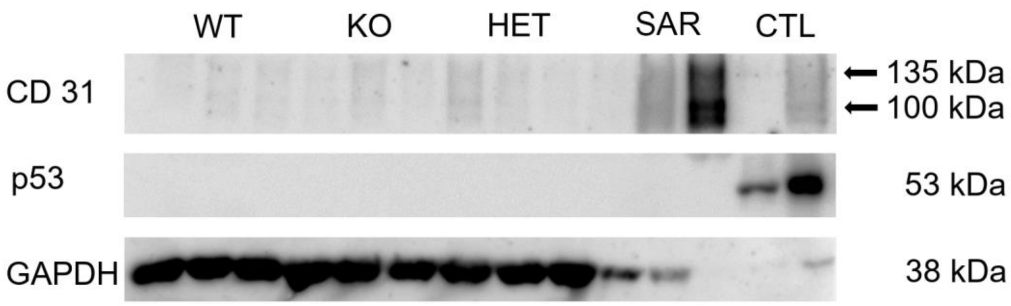

Figure S9. Western blot analysis – p53 and CD31 expression. GAPDH expression was used as a control for protein load. SAR—sarcoma; CTL—positive controls, for CD31 rat white blood cells lysate (left lane) and homogenate from rat aorta (right lane); for p53 lysate from Caki1 cells (left lane) and MDA-MB-231 cells (right lane).

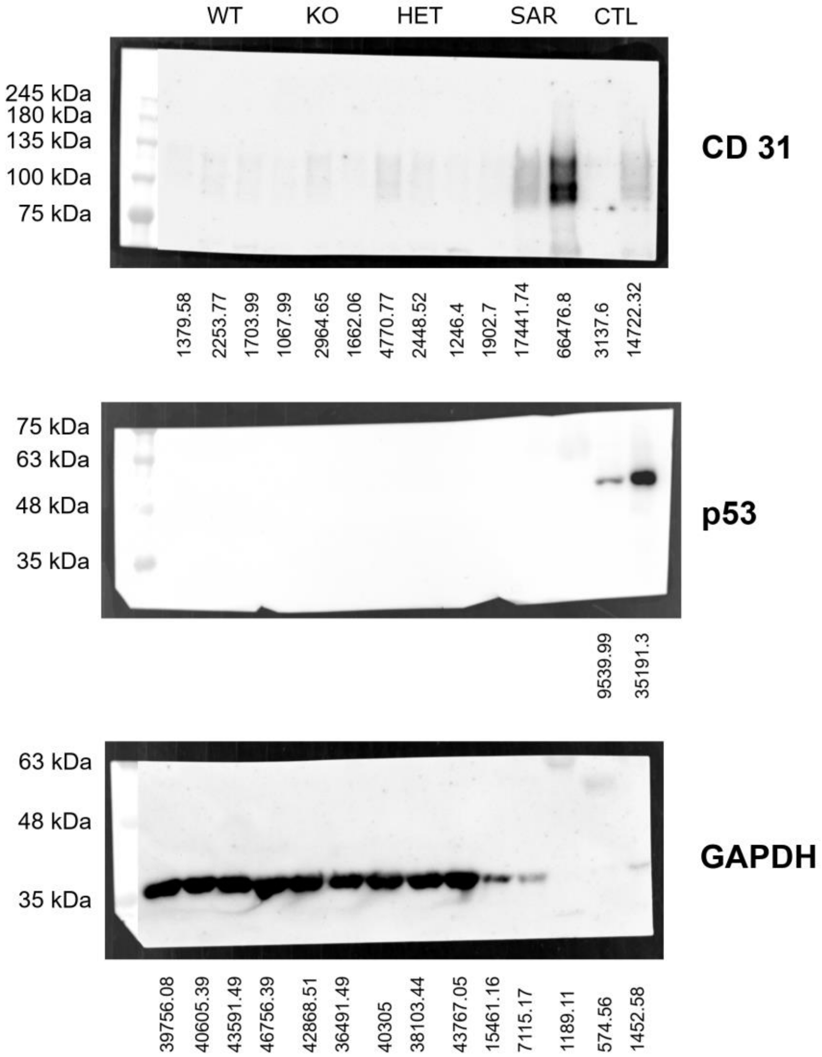

Figure S10. Uncropped combined immunoblotting corresponding to Figure S9 with molecular weight markers and densitometry readings.

© 2020 by the authors. Licensee MDPI, Basel, Switzerland. This article is an open access article distributed under the terms and conditions of the Creative Commons Attribution (CC BY) license (http://creativecommons.org/licenses/by/4.0/).
